# Bis[4-amino-*N*-(4-methyl­pyrimidin-2-yl-κ*N* ^3^)benzene­sulfonamidato-κ*N*](2,2′-bi­pyridine-κ^2^ *N*,*N*′)mercury(II)

**DOI:** 10.1107/S1600536814004760

**Published:** 2014-03-08

**Authors:** G. M. Golzar Hossain, A. J. Amoroso

**Affiliations:** a Department of Chemistry, University of Dhaka, Dhaka 1000, Bangladesh; b School of Chemistry, Cardiff University, Cardiff CF10 3AT, Wales

## Abstract

The complete mol­ecule of the title complex, [Hg(C_11_H_11_N_4_O_2_S)_2_(C_10_H_8_N_2_)], is generated by crystallographic twofold symmetry, with the mercury cation lying on the rotation axis. The mercury coordination polyhedron can be described as tetra­hedral (from the *N*,*N*′-bidenate bi­pyridine mol­ecule and the sulfonamide N atoms of the sulfamerazine anions) or as squashed trigonal-prismatic, if two long (> 2.80 Å) Hg—N bonds to pyrimidine N atoms are included. The dihedral angle between the aromatic rings in the anion is 73.3 (2)°. In the crystal, N—H⋯(N,O) and N—H⋯N hydrogen bonds link the mol­ecules into a three-dimensional network.

## Related literature   

For related structures, see: Garcia-Raso *et al.* (1997 ▶, 2000 ▶); Saladini *et al.* (2001 ▶); Zamora *et al.* (1982 ▶); Hergold-Brundić *et al.* (1989 ▶). For ligand conformations, see: Hossain & Amoroso (2007 ▶, 2012 ▶); Hossain *et al.* (2007 ▶).
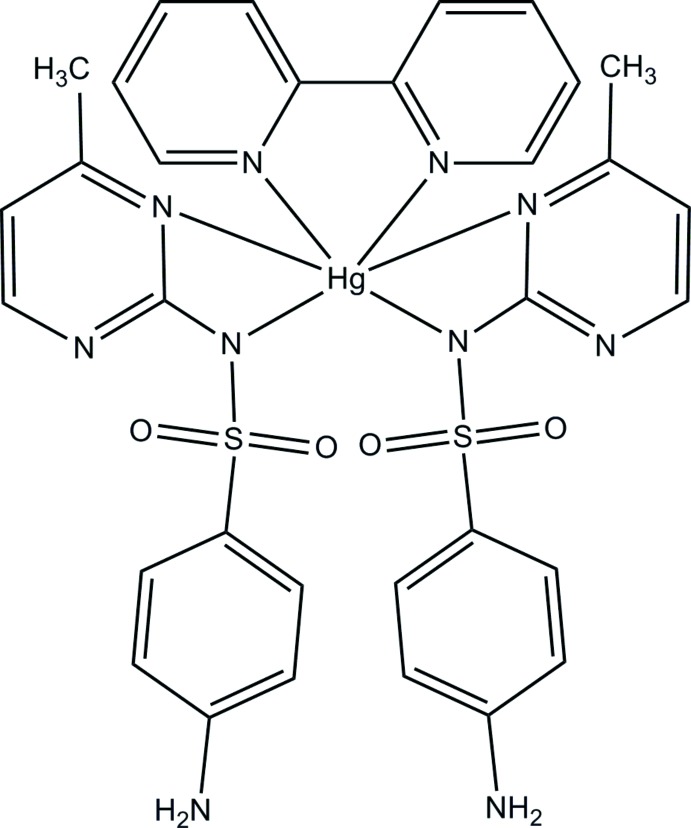


## Experimental   

### 

#### Crystal data  

[Hg(C_11_H_11_N_4_O_2_S)_2_(C_10_H_8_N_2_)]
*M* *_r_* = 883.37
Monoclinic, 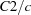

*a* = 18.7483 (8) Å
*b* = 15.0824 (7) Å
*c* = 12.1143 (6) Å
β = 100.202 (2)°
*V* = 3371.4 (3) Å^3^
*Z* = 4
Mo *K*α radiation
μ = 4.74 mm^−1^
*T* = 150 K
0.12 × 0.12 × 0.10 mm

#### Data collection  

Nonius KappaCCD diffractometer
Absorption correction: multi-scan (*SORTAV*; Blessing, 1995 ▶)] *T*_min_ = 0.600, *T*_max_ = 0.648
29910 measured reflections
3874 independent reflections
3408 reflections with *I* > 2σ(*I*)
*R*_int_ = 0.072

#### Refinement  

*R*[*F* ^2^ > 2σ(*F* ^2^)] = 0.039
*wR*(*F* ^2^) = 0.074
*S* = 1.10
3874 reflections
231 parameters
3 restraints
H atoms treated by a mixture of independent and constrained refinement
Δρ_max_ = 1.13 e Å^−3^
Δρ_min_ = −0.75 e Å^−3^

### 

Data collection: *DENZO* (Otwinowski & Minor, 1997 ▶) and *COLLECT* (Hooft, 1998 ▶); cell refinement: *DENZO* and *COLLECT*; data reduction: *DENZO* and *COLLECT*; program(s) used to solve structure: *SHELXS97* (Sheldrick, 2008 ▶); program(s) used to refine structure: *SHELXL2013* (Sheldrick, 2008 ▶); molecular graphics: *ORTEP-3 for Windows* (Farrugia, 2012 ▶); software used to prepare material for publication: *WinGX* (Farrugia, 2012 ▶).

## Supplementary Material

Crystal structure: contains datablock(s) I, global. DOI: 10.1107/S1600536814004760/hb7201sup1.cif

Structure factors: contains datablock(s) I. DOI: 10.1107/S1600536814004760/hb7201Isup2.hkl

CCDC reference: 989386

Additional supporting information:  crystallographic information; 3D view; checkCIF report

## Figures and Tables

**Table 1:** Selected bond lengths (Å)

Hg1—N11	2.214 (4)
Hg1—N1	2.322 (3)
Hg1—N12	2.883 (3)

**Table 2:** Hydrogen-bond geometry (Å, °)

*D*—H⋯*A*	*D*—H	H⋯*A*	*D*⋯*A*	*D*—H⋯*A*
N14—H14*A*⋯O11^i^	0.90 (1)	2.41 (3)	3.173 (5)	143 (4)
N14—H14*A*⋯N13^i^	0.90 (1)	2.42 (3)	3.125 (6)	135 (4)
N14—H14*B*⋯O11^ii^	0.90 (1)	2.13 (1)	2.997 (5)	163 (4)

Symmetry codes: (i) 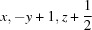; (ii) 
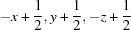
.

**Fig. 1.**
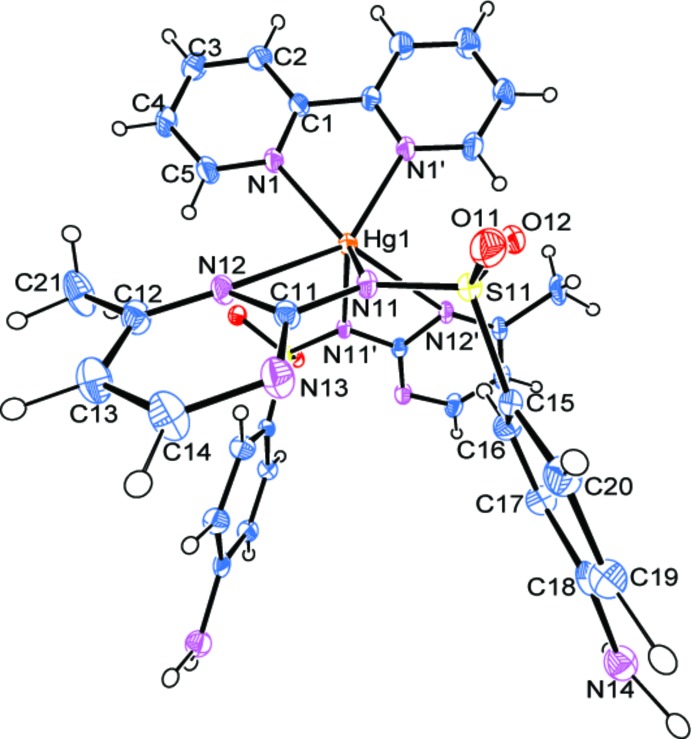
The molecular structure of (I), with 50% probability displacement ellipsoids for non-H atoms.

**Fig. 2.**
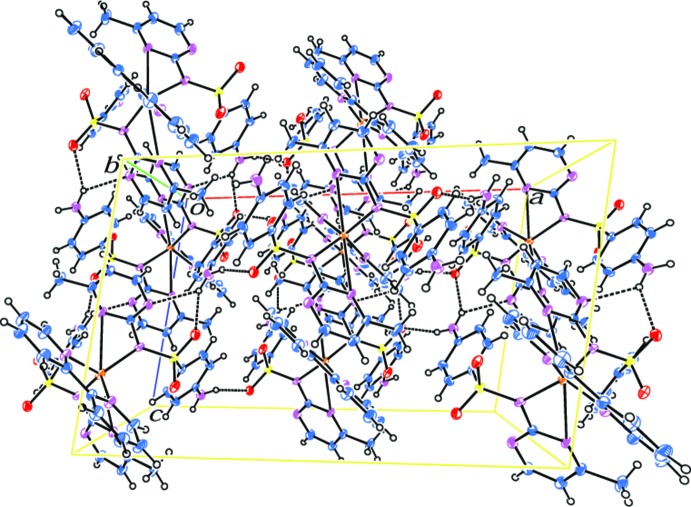
The packing of (I), viewed down the a-axis, showing one layer of molecules connected by N—H···N and N—H···O hydrogen bonds (dashed lines).

## supplementary crystallographic information

### 1. Comment

The mercury complexes of some related ligands have been reported (Garcia-Raso *et al.*, 1997; Hossain *et al.*, 2007). In the title complex, sulfamerazine behaves as a bidentate anionic ligand and Hg(II) ion is coordinated *via* sulfonamidic N(11) and pyrimido N(12) atoms, the fifth and sixth coordination sites are occupied by bipyridine molecule. The geometry of the complex is trigonal prismatic with the Hg atom lying on a twofold axis as the bpy molecule lies on the one part and the two larger sulfamerazine molecules lie on the other part.

The Hg–N(11) bond distance of 2.216 (3) Å is slightly longer than the values of 2.087 (4) Å (Garcia-Raso *et al.*, 1997), 2.071 (4) Å (Garcia-Raso *et al.*, 2000) and 2.14 (2) Å (Saladini *et al.*, 2001). The pyrimido nitrogen [N(12)] atom makes bond with longer interaction with the distance of 2.881 (3) Å. The C(18)—N(14) bond distance of 1.373 (5) Å is in good agreement with the corresponding bond in free sulfamerazine suggesting the terminal amino group is not coordinated with the Hg atoms. The Hg—N(1) bond distance of 2.323 (3) Å is longer than the corresponding bond lengths of 2.140 (3) and 2.124 (3) Å (Zamora *et al.*, 1982) and 2.130 Å (Hergold-Brundić *et al.*, 1989).

The bond angles around the S atom correspond to a distorted tetrahedral geometry. The bond distance C(18)—N(14) (Terminal amino group) of 1.371 (5) Å and the torsion angle C(15)—S(11)—N(11)—C(11) of 74.4 (4)° are larger than those observed in the related structures (Hossain & Amoroso, 2007; Hossain & Amoroso, 2012). The dihedral angle between the aromatic rings of the sulfamerazinate anion of 73.27 (13)° is larger than the value of 71.10 (14)° (Hossain & Amoroso, 2007) and smaller than the value of 76.60 (8)° (Hossain & Amoroso, 2012) in the sulfadiazinate anion.

The packing of the complex unit (Fig. 2) is governed by weak (intermolecular N—H···N, and N—H···O) hydrogen bonds (Table 2) between the amino (–NH2) group of one complex unit and the sulfonyl oxygen and the pyrimido nitrogen atoms of the next units.

### 2. Experimental

The complex was obtained by dissolving (2.723 g, 2 mmol) sulfamerazine in 50 ml of methanol followed by drop-wise addition of Hg(CH_3_COO)_2_·4H_2_O (1.245 g, 1 mmol) in water with constant stirring on hot plate for 1 h. A white precipitate was formed, filtered and washed with methanol and dried in a desiccator over silica gel. The precipitate was dissolved in DMF, bipyri dine (0.235 g, 1 mmol) was added to this solution and stirred for 30 minutes. The solution was filtered and left for crystallization. A week later, colourless blocks were obtained.

### 3. Refinement

The H atoms were positioned geometrically and refined using a riding model except that for terminal amino group N(14) which were located from the difference map and fixed to 0.900 (3) Å], with C—H = 0.95–0.98 Å and with *U*_iso_(H) = 1.2 (1.5 for methyl groups) times *U*_eq_(C).

### Crystal data

**Table:**

[Hg(C_11_H_11_N_4_O_2_S)_2_(C_10_H_8_N_2_)]	*F*(000) = 1744
*M**_r_* = 883.37	*D*_x_ = 1.740 Mg m^−^^3^
Monoclinic, *C*2/*c*	Mo *K*α radiation, λ = 0.71073 Å
*a* = 18.7483 (8) Å	Cell parameters from 3874 reflections
*b* = 15.0824 (7) Å	θ = 2.9–27.5°
*c* = 12.1143 (6) Å	µ = 4.74 mm^−^^1^
β = 100.202 (2)°	*T* = 150 K
*V* = 3371.4 (3) Å^3^	Block, colorless
*Z* = 4	0.12 × 0.12 × 0.10 mm

### Data collection

**Table:**

Nonius KappaCCD diffractometer	3408 reflections with *I* > 2σ(*I*)
Radiation source: fine-focus sealed tube	*R*_int_ = 0.072
ω scans	θ_max_ = 27.5°, θ_min_ = 3.2°
Absorption correction: multi-scan (*SORTAV*; Blessing, 1995)]	*h* = −24→24
*T*_min_ = 0.600, *T*_max_ = 0.648	*k* = −19→19
29910 measured reflections	*l* = −15→15
3874 independent reflections	

### Refinement

**Table:**

Refinement on *F*^2^	3 restraints
Least-squares matrix: full	Hydrogen site location: mixed
*R*[*F*^2^ > 2σ(*F*^2^)] = 0.039	H atoms treated by a mixture of independent and constrained refinement
*wR*(*F*^2^) = 0.074	*w* = 1/[σ^2^(*F*_o_^2^) + (0.0108*P*)^2^ + 13.7149*P*] where *P* = (*F*_o_^2^ + 2*F*_c_^2^)/3
*S* = 1.10	(Δ/σ)_max_ < 0.001
3874 reflections	Δρ_max_ = 1.13 e Å^−^^3^
231 parameters	Δρ_min_ = −0.75 e Å^−^^3^

### Special details

**Table:**

Geometry. All e.s.d.'s (except the e.s.d. in the dihedral angle between two l.s. planes) are estimated using the full covariance matrix. The cell e.s.d.'s are taken into account individually in the estimation of e.s.d.'s in distances, angles and torsion angles; correlations between e.s.d.'s in cell parameters are only used when they are defined by crystal symmetry. An approximate (isotropic) treatment of cell e.s.d.'s is used for estimating e.s.d.'s involving l.s. planes.

### Fractional atomic coordinates and isotropic or equivalent isotropic displacement parameters (Å^2^)

**Table:**

	*x*	*y*	*z*	*U*_iso_*/*U*_eq_	
Hg1	0.0000	0.15586 (2)	0.2500	0.02712 (9)	
S11	0.14988 (6)	0.26641 (7)	0.20322 (9)	0.0241 (2)	
O11	0.19225 (16)	0.26047 (19)	0.1139 (2)	0.0300 (7)	
O12	0.17100 (16)	0.20881 (18)	0.2977 (2)	0.0298 (7)	
N11	0.0655 (2)	0.2427 (2)	0.1595 (3)	0.0250 (8)	
N12	−0.0384 (2)	0.2333 (2)	0.0300 (3)	0.0272 (8)	
N13	0.0520 (2)	0.3400 (2)	0.0030 (3)	0.0321 (9)	
N14	0.1869 (2)	0.6308 (2)	0.3883 (3)	0.0297 (9)	
C11	0.0260 (2)	0.2745 (3)	0.0597 (3)	0.0236 (9)	
C12	−0.0808 (2)	0.2607 (3)	−0.0646 (4)	0.0316 (10)	
C13	−0.0589 (3)	0.3277 (3)	−0.1283 (4)	0.0382 (12)	
H13	−0.0889	0.3474	−0.1954	0.046*	
C14	0.0076 (3)	0.3648 (3)	−0.0915 (4)	0.0390 (12)	
H14	0.0233	0.4107	−0.1353	0.047*	
C15	0.1581 (2)	0.3761 (3)	0.2533 (3)	0.0228 (9)	
C16	0.1290 (3)	0.3989 (3)	0.3461 (4)	0.0304 (10)	
H16	0.1012	0.3566	0.3783	0.036*	
C17	0.1398 (3)	0.4825 (3)	0.3930 (4)	0.0292 (10)	
H17	0.1204	0.4965	0.4581	0.035*	
C18	0.1790 (2)	0.5466 (3)	0.3453 (3)	0.0254 (9)	
C19	0.2096 (2)	0.5225 (3)	0.2518 (4)	0.0290 (10)	
H19	0.2379	0.5644	0.2197	0.035*	
C20	0.1989 (2)	0.4381 (3)	0.2058 (4)	0.0289 (10)	
H20	0.2193	0.4226	0.1421	0.035*	
C21	−0.1540 (3)	0.2183 (4)	−0.0972 (5)	0.0514 (15)	
H21A	−0.1481	0.1554	−0.1135	0.077*	
H21B	−0.1807	0.2478	−0.1641	0.077*	
H21C	−0.1811	0.2241	−0.0354	0.077*	
N1	−0.0594 (2)	0.0298 (2)	0.1713 (3)	0.0302 (8)	
C1	−0.0330 (2)	−0.0493 (3)	0.2060 (3)	0.0275 (10)	
C2	−0.0665 (3)	−0.1265 (3)	0.1612 (4)	0.0381 (12)	
H2	−0.0474	−0.1826	0.1872	0.046*	
C3	−0.1274 (3)	−0.1219 (3)	0.0792 (4)	0.0435 (13)	
H3	−0.1510	−0.1744	0.0485	0.052*	
C4	−0.1535 (3)	−0.0397 (3)	0.0424 (4)	0.0423 (12)	
H4	−0.1944	−0.0344	−0.0160	0.051*	
C5	−0.1189 (3)	0.0351 (3)	0.0922 (4)	0.0400 (12)	
H5	−0.1379	0.0919	0.0696	0.048*	
H14A	0.171 (2)	0.644 (3)	0.452 (2)	0.038 (14)*	
H14B	0.2224 (18)	0.666 (2)	0.373 (4)	0.040 (14)*	

### Atomic displacement parameters (Å^2^)

**Table:**

	*U*^11^	*U*^22^	*U*^33^	*U*^12^	*U*^13^	*U*^23^
Hg1	0.02493 (15)	0.02381 (12)	0.02946 (13)	0.000	−0.00387 (9)	0.000
S11	0.0190 (6)	0.0238 (5)	0.0276 (5)	0.0009 (4)	−0.0009 (4)	−0.0021 (4)
O11	0.0231 (18)	0.0329 (16)	0.0338 (16)	−0.0007 (13)	0.0046 (14)	−0.0080 (14)
O12	0.0218 (17)	0.0271 (15)	0.0366 (17)	0.0026 (13)	−0.0053 (14)	0.0021 (13)
N11	0.025 (2)	0.0279 (18)	0.0192 (16)	0.0003 (15)	−0.0048 (15)	−0.0001 (15)
N12	0.017 (2)	0.0334 (19)	0.0283 (19)	0.0025 (15)	−0.0028 (15)	−0.0003 (16)
N13	0.025 (2)	0.039 (2)	0.0312 (19)	−0.0006 (18)	0.0009 (16)	0.0055 (18)
N14	0.030 (2)	0.0259 (19)	0.033 (2)	−0.0040 (16)	0.0056 (18)	−0.0031 (16)
C11	0.017 (2)	0.028 (2)	0.025 (2)	0.0039 (17)	0.0028 (17)	−0.0006 (18)
C12	0.018 (2)	0.041 (3)	0.033 (2)	0.0021 (19)	−0.0034 (19)	−0.002 (2)
C13	0.027 (3)	0.053 (3)	0.032 (2)	0.004 (2)	−0.004 (2)	0.006 (2)
C14	0.031 (3)	0.049 (3)	0.034 (3)	0.002 (2)	0.002 (2)	0.014 (2)
C15	0.018 (2)	0.0217 (19)	0.026 (2)	−0.0015 (16)	−0.0024 (17)	−0.0016 (17)
C16	0.028 (3)	0.031 (2)	0.034 (2)	−0.0052 (19)	0.010 (2)	0.002 (2)
C17	0.033 (3)	0.031 (2)	0.026 (2)	−0.0009 (19)	0.011 (2)	−0.0022 (19)
C18	0.017 (2)	0.030 (2)	0.026 (2)	−0.0009 (17)	−0.0057 (17)	0.0007 (18)
C19	0.026 (3)	0.028 (2)	0.033 (2)	−0.0038 (19)	0.006 (2)	0.0015 (19)
C20	0.026 (3)	0.032 (2)	0.029 (2)	−0.0007 (19)	0.0073 (19)	−0.0029 (19)
C21	0.023 (3)	0.076 (4)	0.047 (3)	−0.005 (3)	−0.014 (2)	0.020 (3)
N1	0.027 (2)	0.0289 (18)	0.0315 (19)	−0.0013 (16)	−0.0045 (17)	−0.0004 (16)
C1	0.025 (3)	0.030 (2)	0.028 (2)	0.0036 (18)	0.0033 (19)	0.0007 (19)
C2	0.042 (3)	0.025 (2)	0.046 (3)	−0.001 (2)	0.001 (2)	−0.007 (2)
C3	0.043 (3)	0.036 (3)	0.048 (3)	−0.009 (2)	−0.002 (3)	−0.015 (2)
C4	0.035 (3)	0.046 (3)	0.039 (3)	−0.008 (2)	−0.011 (2)	−0.010 (2)
C5	0.034 (3)	0.037 (3)	0.044 (3)	0.002 (2)	−0.010 (2)	0.001 (2)

### Geometric parameters (Å, º)

**Table:**

Hg1—N11	2.214 (4)	C15—C20	1.394 (6)
Hg1—N11^i^	2.214 (4)	C16—C17	1.383 (6)
Hg1—N1	2.322 (3)	C16—H16	0.9500
Hg1—N1^i^	2.322 (3)	C17—C18	1.400 (6)
Hg1—N12^i^	2.883 (3)	C17—H17	0.9500
Hg1—N12	2.883 (3)	C18—C19	1.405 (6)
S11—O12	1.436 (3)	C19—C20	1.390 (6)
S11—O11	1.454 (3)	C19—H19	0.9500
S11—N11	1.616 (4)	C20—H20	0.9500
S11—C15	1.760 (4)	C21—H21A	0.9800
N11—C11	1.387 (5)	C21—H21B	0.9800
N12—C12	1.339 (5)	C21—H21C	0.9800
N12—C11	1.349 (5)	N1—C1	1.331 (5)
N13—C11	1.343 (6)	N1—C5	1.337 (6)
N13—C14	1.344 (6)	C1—C2	1.387 (6)
N14—C18	1.371 (5)	C1—C1^i^	1.483 (9)
N14—H14A	0.900 (3)	C2—C3	1.376 (7)
N14—H14B	0.900 (3)	C2—H2	0.9500
C12—C13	1.378 (7)	C3—C4	1.378 (7)
C12—C21	1.503 (7)	C3—H3	0.9500
C13—C14	1.367 (7)	C4—C5	1.386 (6)
C13—H13	0.9500	C4—H4	0.9500
C14—H14	0.9500	C5—H5	0.9500
C15—C16	1.378 (6)		
			
N11—Hg1—N11^i^	107.50 (18)	C16—C15—C20	119.7 (4)
N11—Hg1—N1	123.31 (12)	C16—C15—S11	119.5 (3)
N11^i^—Hg1—N1	114.81 (13)	C20—C15—S11	120.6 (3)
N11—Hg1—N1^i^	114.81 (13)	C15—C16—C17	120.8 (4)
N11^i^—Hg1—N1^i^	123.31 (12)	C15—C16—H16	119.6
N1—Hg1—N1^i^	70.00 (17)	C17—C16—H16	119.6
N11—Hg1—N12^i^	98.53 (11)	C16—C17—C18	120.7 (4)
N11^i^—Hg1—N12^i^	51.11 (11)	C16—C17—H17	119.7
N1—Hg1—N12^i^	137.29 (11)	C18—C17—H17	119.7
N1^i^—Hg1—N12^i^	85.95 (11)	N14—C18—C17	120.8 (4)
N11—Hg1—N12	51.11 (11)	N14—C18—C19	120.9 (4)
N11^i^—Hg1—N12	98.53 (11)	C17—C18—C19	118.3 (4)
N1—Hg1—N12	85.95 (11)	C20—C19—C18	120.6 (4)
N1^i^—Hg1—N12	137.29 (11)	C20—C19—H19	119.7
N12^i^—Hg1—N12	132.17 (14)	C18—C19—H19	119.7
O12—S11—O11	116.49 (18)	C19—C20—C15	120.0 (4)
O12—S11—N11	104.05 (18)	C19—C20—H20	120.0
O11—S11—N11	111.99 (18)	C15—C20—H20	120.0
O12—S11—C15	107.41 (18)	C12—C21—H21A	109.5
O11—S11—C15	106.76 (19)	C12—C21—H21B	109.5
N11—S11—C15	110.01 (19)	H21A—C21—H21B	109.5
C11—N11—S11	123.3 (3)	C12—C21—H21C	109.5
C11—N11—Hg1	112.3 (3)	H21A—C21—H21C	109.5
S11—N11—Hg1	124.42 (18)	H21B—C21—H21C	109.5
C12—N12—C11	117.0 (4)	C1—N1—C5	119.8 (4)
C12—N12—Hg1	158.4 (3)	C1—N1—Hg1	118.7 (3)
C11—N12—Hg1	82.8 (2)	C5—N1—Hg1	121.6 (3)
C11—N13—C14	114.5 (4)	N1—C1—C2	120.7 (4)
C18—N14—H14A	120 (3)	N1—C1—C1^i^	116.3 (2)
C18—N14—H14B	120 (3)	C2—C1—C1^i^	122.9 (3)
H14A—N14—H14B	115 (3)	C3—C2—C1	120.0 (4)
N13—C11—N12	126.2 (4)	C3—C2—H2	120.0
N13—C11—N11	121.0 (4)	C1—C2—H2	120.0
N12—C11—N11	112.8 (4)	C2—C3—C4	118.8 (4)
N12—C12—C13	121.0 (4)	C2—C3—H3	120.6
N12—C12—C21	118.1 (4)	C4—C3—H3	120.6
C13—C12—C21	120.9 (4)	C3—C4—C5	118.6 (4)
C14—C13—C12	117.6 (4)	C3—C4—H4	120.7
C14—C13—H13	121.2	C5—C4—H4	120.7
C12—C13—H13	121.2	N1—C5—C4	122.0 (4)
N13—C14—C13	123.8 (5)	N1—C5—H5	119.0
N13—C14—H14	118.1	C4—C5—H5	119.0
C13—C14—H14	118.1		

Symmetry code: (i) −*x*, *y*, −*z*+1/2.

### Hydrogen-bond geometry (Å, º)

**Table:**

*D*—H···*A*	*D*—H	H···*A*	*D*···*A*	*D*—H···*A*
N14—H14*A*···O11^ii^	0.90 (1)	2.41 (3)	3.173 (5)	143 (4)
N14—H14*A*···N13^ii^	0.90 (1)	2.42 (3)	3.125 (6)	135 (4)
N14—H14*B*···O11^iii^	0.90 (1)	2.13 (1)	2.997 (5)	163 (4)

Symmetry codes: (ii) *x*, −*y*+1, *z*+1/2; (iii) −*x*+1/2, *y*+1/2, −*z*+1/2.
